# Prostaglandins and prostaglandin receptor antagonism in migraine

**DOI:** 10.1186/1129-2377-14-S1-P114

**Published:** 2013-02-21

**Authors:** M Antonova

**Affiliations:** 1 Danish Headache Center, Denmark

## 

Human models of headache may contribute to understanding of prostaglandins’ role in migraine pathogenesis. The current thesis investigated the migraine triggering effect of prostaglandin E2 (PGE2) in migraine patients without aura, the efficacy of a novel EP4 receptor antagonist, BGC20- 1531, in prevention of PGE2-induced headache and whether vasoconstricting and pro-nociceptive prostaglandin F2α (PGF2α) triggers headache in healthy volunteers. All studies were designed as double-blind, placebo-controlled, cross-over experiments. PGE2 /PGF2α or saline were infused over 20-25 min. In study with EP4 receptor antagonist healthy volunteers were pre-treated with two different doses of BGC20-1531 or placebo followed by PGE2 infusion over 25 min. The headache data were collected during the whole study dag, whereas the possible vascular changes were measured during the in-hospital phase of 1.5 h. The infusion of PGE2 caused the immediate migraine-like attacks and vasodilatation of the middle cerebral artery in migraine patients without aura. A highly specific and potent EP4 receptor antagonist, BGC20-1531, was not able to attenuate PGE2-induced headache and vasodilatation of both intra- and extra-cerebral arteries in healthy volunteers [1]. Intravenous infusion of PGF2α did not induce headache or statistically significant vasoconstriction of cerebral arteries in healthy subjects [2]. PGE2 provoked immediate migraine-like attacks in patients with migraine without aura. These data suggest that PGE2 may play an important role in the pathogenesis of migraine. The lack of efficacy of EP4 receptor antagonist suggests that a single receptor blockade is not sufficient to block PGE2 induced responses; hence EP2 receptor should be investigated as a potential drug target for the treatment of migraine. The absence of headache during the PGF2α infusion indicates that vasodilating properties are necessary for the induction of headache and migraine after prostanoids.

## References

[B1] Antonova M Wienecke T Maubach K The pharmacological effect of BGC20-1531, a novel prostanoid EP(4) receptor antagonist, in the Prostaglandin E (2) human model of headache J Headache Pain 2011 14 551 559 10.1007/s10194-011-0358-9 21681585 PMC3173651

[B2] Antonova M Wienecke T Olesen J Pro-inflammatory and vasoconstricting prostanoid PGF2á causes no headache in man Cephalalgia 2011 14 15 1532 1541 10.1177/0333102411423314 22013143

